# Subjective health expectations of patients with age-related macular degeneration treated with antiVEGF drugs

**DOI:** 10.1186/s12877-017-0619-9

**Published:** 2017-10-10

**Authors:** Márta Péntek, Valentin Brodszky, Zsolt Biró, Zsófia Kölkedi, Árpád Dunai, János Németh, Petra Baji, Fanni Rencz, László Gulácsi, Miklós D. Resch

**Affiliations:** 1 Department of Health Economics, Corvinus University of Budapest, Fővám tér 8, Budapest, 1093 Hungary; 2 Department of Ophthalmology, Pécs University of Sciences, Nyár u. 8, Pécs, 7624 Hungary; 3 Department of Ophthalmology, Semmelweis University Budapest, Mária u. 39, Budapest, 1085 Hungary; 4 Clinical Medicine Doctoral School, Semmelweis University Budapest, Ulloi u. 26, Budapest, 1085 Hungary

**Keywords:** Age-related macular degeneration, Health-related quality of life, EQ-5D, Time trade-off, Subjective life-expectancy, Health expectations

## Abstract

**Background:**

Subjective expectations regarding future health may influence patients’ judgement of current health and treatment effects, as well as adherence to therapies in chronic diseases. We aimed to explore subjective expectations on longevity and future health-related quality of life (HRQOL) of patients with age-related macular degeneration (AMD) treated with antiVEGF injections and analyse the influencing factors.

**Methods:**

Consecutive AMD patients in two ophthalmology centres were included. Demographics, clinical characteristics and informal care utilisation were recorded. Current health was evaluated by the EQ-5D generic health status questionnaire and time trade-off (TTO) methods. Happiness was measured on a visual analogue scale (VAS). Subjective life-expectancy and expected EQ-5D status at ages 70, 80 and 90 were surveyed. T-test was applied to compare subgroups and Pearson correlations were performed to analyse relationships between variables.

**Results:**

One hundred twenty two patients were involved (females 62%) with a mean (SD) age of 75.2 (7.9) years and disease duration of 2.9 (2.5) years. The majority were in AREDS-4 state, the better eye’s ETDRS was 64.7 (15.4). EQ-5D and TTO revealed moderate deterioration of health (0.66 vs. 0.72, *p* = 0.131), happiness VAS was 6.3 (2.2). Correlation between EQ-5D and ETDRS was moderate (*R* = 0.242, *p* < 0.05) and having both versus one eye in AREDS-4 resulted lower TTO (0.68 vs. 0.83; *p* = 0.013). Subjective life-expectancy did not differ significantly from statistical life-expectancy and had no significant impact on TTO. The self-estimated mean EQ-5D score was 0.60, 0.40 and 0.24 for ages 70, 80 and 90 which is lower than the population norm of age-groups 65–74, 75–84 and 85+ (0.77, 0.63 and 0.63, respectively). Age, gender, current EQ-5D, need for informal care and happiness were deterministic factors of subjective health expectations.

**Conclusion:**

AMD patients with antiVEGF treatment have comparable HRQOL as the age-matched general public but expect a more severe deterioration of health with age. Older patients with worse HRQOL have worse subjective expectations. Exploring patients’ health expectations provides an opportunity for ophthalmologists to correct misperceptions and improve the quality of AMD care. Further studies should provide evidences on the relationship between subjective expectations and actual health outcomes, and on its impact on patients’ AMD-specific health behaviour.

**Electronic supplementary material:**

The online version of this article (10.1186/s12877-017-0619-9) contains supplementary material, which is available to authorized users.

## Background

Age-related macular degeneration (AMD) is one of the main causes of vision loss in adulthood in developed countries [1]. People with AMD often develop other health issues as well, patients often suffer from depression which has a negative impact on their health [2, 3]. Exudative form (also called wet AMD) resulted in irreversible severe visual impairment until the introduction of the highly effective anti-vascular endothelial growth factor (antiVEGF) biological treatments. Patient-physician collaboration holds promise for improving patient care because antiVEGF therapy requires regular monitoring and re-treatments might be necessary to preserve the beneficial effects [4].

Patient-physician communication about the expected benefits and risks of the antiVEGF therapy as well as to get insight into patient’s preferences and views for their future health is an integral part of the clinical practice [5]. However, subjective expectations are rarely observed. One way to overcome the problem is to elicit future health beliefs of individuals, or so-called subjective health expectations, directly from the patients by surveys. All AMD patients live with some uncertainty about the course of the disease, future health, including longevity, health related quality of life (HRQL) and happiness [6]. Happiness is considered as a subjective measure of the overall satisfaction of life, generally defined as ‘the degree to which an individual judges the overall quality of his life favourably’ and is frequently designated as an important life goal [7–9].

Some patients anticipate a great discomfort related to the antiVEGF treatment and experience the intravitreal injections as stressful events, especially in the beginning of the treatment [10, 11]. Comparing and valuing this short term burden and expected long term health benefits from the antiVEGF treatment might influence patients’ participation in AMD care and treatment adherence. Previous studies including the general population revealed that individuals tend to overestimate their life-expectancy and underestimate their health-related quality of life (HRQOL) for future ages [12, 13]. Age and current health state were found to be important explanatory factors for subjective health expectations. Brouwer and van Exel highlighted the importance of subjective health expectations studies given the influence inaccurate expectations may have on actual health behaviour, treatment participation in health care and acceptance of lifestyle changes [12]. Exploring patient’s subjective health expectations has an increasing role in clinical decision-making, however no study results available in AMD.

Subjective health expectations are important from the health economics aspect as well. Highly effective but costly biological therapies have increased interest in the economic aspects of AMD treatments [4, 14–18]. To conduct a proper economic analysis, data on the value of different health states expressed in utility are required. Utilities are used to calculate the quality-adjusted life year (QALY) gains of different treatment strategies in cost-effectiveness analyses. Health state utility values in AMD were analysed in two recent systematic literature reviews and the time trade-off (TTO) method was found to be a suitable health state valuation technique in AMD [19, 20]. The TTO consists of a hypothetical trade-off in which subjects are asked to indicate the number of life-years they would be willing to sacrify to avoid a well-defined poorer health state [21–23]. Subjective expectations on longevity and future health may influence TTO responses [24].

In this paper we present findings on subjective health expectations of patients with AMD treated with antiVEGF biologicals in Hungary. We assess patients’ HRQOL including two health state utility measures, namely the EQ-5D and TTO [25]. We analyse the correlations among subjective health expectations, patients’ current visual status and HRQOL, patients’ dependence on others and patients’ happiness.

## Methods

### Study design and patients

We conducted a non-interventional, cross-sectional questionnaire survey in two university ophthalmology clinics in Hungary, involving patients and their ophthalmologists. Patients with diagnosis of wet AMD on at least one eye confirmed by ophthalmologist, aged ≥ 18 years and who were treated with or were eligible to begin biological intravitreal treatment were invited to participate in the study. Recruitment run between November of 2012 and December of 2013 and patients who gave written informed consent to participate in the study were consecutively enrolled. The study was performed in accordance with the Declaration of Helsinki. Ethical approval was obtained from the national ethical committee (ETT–TUKEB7946/2013/EKU).

### Questionnaire survey

The questionnaire consisted of two main sections: the first section was completed by the patients and the second was filled by their ophthalmologists (Additional file 1). Patients completed a set of questions in which socio-economic data, health care utilisation, self-assessed disease activity on Visual Function Index-14 (VF-14), health related quality of life measured by EQ-5D questionnaire, and utility of the current health status assessed by time-trade-off (TTO) and happiness were surveyed. (See next sections.)

AMD related clinical data (date of diagnosis, vision by Early Treatment Diabetic Retinopathy Study - ETDSR, Age-Related Eye Disease Study - AREDS state and therapies in the past 12 months, current treatment) and comorbidities were provided by ophthalmologists [26, 27].

### Self-completed questionnaire survey - the patients

Patients who had difficulties with reading due to visual problems were helped by an interviewer who read the questions and recorded answers. Demographics and employment status was surveyed, as well as the number of AMD related hospitalizations in the previous 12 months and informal care due to AMD was assessed for the past month (the number of hours per week provided by others to help the patient in his/her everyday activities).

A Hungarian translation of the Visual Function Index-14 (VF-14) was used to assess disease-specific functional status. The VF-14 ranges between 0 (not affected) to 100 (extremely affected), the higher scores correspond to a higher impairment [28].

### Current health related quality of life (HRQOL): EQ-5D, time-trade-off (TTO) and happiness

We applied two methods to assess patients’ current HRQOL, both provide utility values on the current health state: EQ-5D and TTO.

The validated Hungarian version of the EQ-5D questionnaire was completed by the patients which comprises a descriptive system (EQ-5D-3 L) and a Visual Analogue Scale (EQ VAS, with endpoints labelled as ‘Worst imaginable health state’ and ‘Best imaginable health state’, range 0–100) [25]. The descriptive system distinguishes between five health domains (mobility, self-care, usual activities, pain/discomfort, and anxiety/depression), and three health state levels for each health domain corresponding to no problems, some problems, and severe problems. The patient was asked to indicate his/her current health state in each of the five dimensions. A HRQOL utility score (the EQ-5D score), as valued by the general public, was calculated from the patient reported health status on the descriptive system. Due to lack of national dataset the UK tariffs were applied for the calculation (range -0.594 – 1).

Time trade-off (TTO) is a broadly used method to measure health state utility directly among patients [29–31].

First, patients were asked to indicate the age they expected to reach. In the next question, they were asked to estimate how many years they would sacrify of their remaining lifetime in return for perfect vision. Utilities were calculated by the following functions where U denotes utility, r is the self-estimated remaining lifespan and x is sacrificed life-years:

$$ \mathrm{U}=1\hbox{--} \mathrm{x}/\mathrm{r}. $$

For instance, a respondent who traded 2 years of his remaining lifespan of 10 years to live with perfect vision, the utility would be U = 1–2/10 = 0.8.

Patients were asked to value their happiness as well, i.e. how much happy they feel in general. A 0–10 visual analogue scale (VAS) was used in which the two endpoints were defined as ‘very unhappy’ and ‘very happy’.

### Subjective expectations for future HRQOL and longevity

Patients were asked to estimate their expected HRQOL at older ages. To make the expectations comparable to current HRQOL, statements of the EQ-5D descriptive system were applied for the assessment (Additional file 1). Patients had to imagine that they were at the age of 60, 70, 80 and 90 years, respectively, and indicate the level of health deterioration they expected to have at these ages in each domain of the EQ-5D. If patients responded to questions about future life years that they had already reached, answers were excluded. This methodology was previously used in the Netherlands and as well in Hungary in a large expectation survey on the general population, and also in recent Hungarian studies with rheumatoid arthritis and psoriasis patients [12, 13, 32]. Subjective life-expectancy was surveyed as detailed above at the TTO section.

### Analysis of determinants of subjective health expectations

Subgroups were developed by patients’ demographic and clinical characteristics: visual status measures, AMD-related hospitalisation in the past 12 months and informal care use. Subgroups for ‘survivors’ vs. ‘non-survivors’ were also established: subjective HRQOL expectations for ages between 60 and 90 were analyzed for subgroups who believe to reach that age (so called ‘survivors’) and for those who do not (‘non-survivors’). Previous studies revealed that patients’ beliefs on longevity might influence considerably their subjective HRQOL expectations for future ages [12, 13, 32]. These subgroups were used to compare subjective life-expectancy and future HRQOL expectations results.

Subjective expectations on longevity were compared to the gender- and age-matched statistical life expectancy in Hungary [33]. Determinants of over- and underestimation were analyzed. Correlations between current visual status (ETDRS, VF-14) and HRQOL outcomes (EQ-5D, TTO and happiness VAS) were analyzed, as well as their relations with subjective expectations on longevity and future HRQOL. Patients’ age was also considered in this analysis.

### Statistical analysis

Statistical analysis of the data was carried out using SPSS Version 20.0 for Windows. Descriptive statistics were performed, T-test was applied to compare subgroups and Pearson correlations were performed to analyse relationships between variables. The level of significance was set to 0.05.

## Results

### Sample characteristics

Altogether 122 patients completed the questionnaire, 62% were female. Mean age of the patients was 75.2 (SD 7.9, range: 56–90) years and the disease duration was 2.9 (SD 2.5) years. Main characteristics and visual status of the patients are presented in Table 1.

**Table 1:** Main characteristics of patients

Variables	Mean (SD)/N (%)
Males	46 (38)
Age, year	75.2 (7.9)
Highest educational level (*n* = 121)	
- Primary school	37 (31)
- Secondary school	53 (44)
- College (Bsc)	15 (12)
- University (Msc)	16 (13)
Lives in the same household (*n* = 121)	
- Alone	43 (35)
- With others	78 (64)
Residence (*n* = 121)	
- Capital (Budapest)	46 (38)
- County town	16 (13)
- Other town	39 (32)
- Village	20 (17)
Employment status^a^ (*n* = 122)	
- Active, works in a payed job full time	2 (2)
- Active, works in a payed job part time	0 (0)
- Disability pensioner	2 (2)
- Retired	119 (98)
- Unemployed	0 (0)
Comorbidity^b^ (*n* = 122)	
- Parkinson disease	1 (1)
- Diabetes	21 (17)
- Hypertension	82 (67)
- Depression	12 (10)
Visual acuity (ETDRS 0–100)	
- Left eye	49.8 (27.0)
- Right eye	42.6 (29.0)
better eye (greater ETDRS score)	64.7 (15.4)
AREDS categories, left eye	
- AREDS 1	5 (4)
- AREDS 2	1 (1)
- AREDS 3	9 (8)
- AREDS 4	98 (87)
AREDS categories, right eye	
- AREDS 1	8 (7)
- AREDS 2	2 (2)
- AREDS 3	2 (2)
- AREDS 4	104 (89)
Both eyes in AREDS 4	84 (69)

^a^1 retired patient was working

^b^patients could have more than one comorbidities

Twenty-six patients (21%) were admitted to hospital due to AMD in the past 12 months. Altogether 35 patients (29%) required help from others for everyday activities (informal care) due to AMD (Table 2).

**Table 2:** Actual HRQOL and subjective expectations for the future

	N (percent)	Current EQ-5D score	EQ VAS	VF-14	Happiness (0–10)	TTO	Statistical remaining life years**	Expected remaining life years	Expected EQ-5D scores for future ages		
									70	80	90
Response rate, N (%)		122 (100)	122 (100)	122 (100)	122 (100)	107 (88)	122 (100)	108 (89)	33 (27)	66 (54)	69 (57)
Total sample	122	0.66 (0.33)	59 (16)	53 (28)	6.3 (2.2)	0.72 (0.30)	10.2 (4.8)	10.4 (6.6)	0.60 (0.41)	0.40 (0.51)	0.24 (0.53)
Gender											
Female	76 (62)	0.56 (0.35)	57 (16)*	50 (26)	6.1 (2.3)	0.68 (0.30)	10.5 (5.4)	9.9 (6.0)	0.57 (0.40)	0.29 (0.56)*	0.08 (0.54)*
Male	46 (38)	0.81 (0.24)	61 (17)*	58 (31)	6.5 (2.2)	0.79 (0.28)	9.6 (3.4)	11.3 (7.4)	0.65 (0.43)	0.54 (0.41)*	0.51 (0.39)*
AREDS											
One eye in AREDS 4	38 (31)	0.72 (0.28)	61 (18)	57 (33)	6.3 (2.0)	0.83 (0.25)*	10.3 (5.2)	11.3 (7.4)	0.51 (0.55)	0.41 (0.44)	0.24 (0.48)
Both eyes in AREDS 4	84 (69)	0.63 (0.35)	58 (16)	51 (25)	6.2 (2.3)	0.68 (0.31)*	10.1 (4.6)	10.1 (6.2)	0.64 (0.31)	0.39 (0.56)	0.23 (0.53)
ETDRS change > 15											
No change	97 (81)	0.65 (0.33)	58 (16)	53 (27)	6.4 (2.2)	0.73 (0.30)	9.9 (4.8)	10.5 (6.6)	0.60 (0.42)	0.40 (0.50)	0.24 (0.53)
Deterioration	23 (19)	0.71 (0.35)	58 (22)	48 (48)	5.7 (2.0)	0.60 (0.32)	11.3 (5.0)	9.6 (6.8)	0.62 (0.15)	0.39 (0.60)	0.20 (0.66)
Expected survivors N (%)	–	–	–	–	–	–	–	–	29 (97)	33 (54)	23 (39)
Yes***: expected age > future age	–	–	–	–	–	–	–	–	0.57 (0.40)	0.58 (0.37)	0.40 (0.47)
No: expected age < future age		–	–	–	–	–	–	–	0.07 (0)	0.17 (0.59)	0.11 (0.57)
Hospitalization in the last 12 months due to AMD											
No	96 (79)	0.65 (0.33)	57 (16)	55 (26)	6.4 (2.2)	0.70 (0.29)	10.2 (4.7)	10.2 (5.9)	0.63 (0.33)	0.44 (0.52)	0.32 (0.51)*
Yes	26 (21)	0.67 (0.35)	63 (19)	47 (33)	5.9 (2.5)	0.79 (0.33)	9.9 (5.1)	11.5 (8.8)	0.51 (0.64)	0.27 (0.48)	0.04 (0.54)*
Use of home help in the last month (informal care)											
No	87 (71)	0.76 (0.27)*	61 (16)*	59 (28)*	6.5 (2.1)*	0.76 (0.28)*	10.4 (4.5)	10.9 (7.0)	0.69 (0.31)*	0.47 (0.48)*	0.35 (0.50)*
Yes	35 (29)	0.41 (0.36)*	53 (16)*	39 (23)*	5.7 (2.6)*	0.63 (0.32)*	9.5 (5.4)	9.2 (5.1)	0.36 (0.53)*	0.02 (0.54)*	−0.03 (0.51)*
Biological treatment											
Received in the past 12 months	93 (76)	0.66 (0.33)	57 (16)	55 (27)	6.5 (2.2)	0.70 (0.29)	9.9 (4.7)	10.5 (6.4)	0.65 (0.42)	0.49 (0.51)*	0.34 (0.52)*
Eligible	29 (24)	0.65 (0.34)	63(16)	46 (31)	5.7 (2.3)	0.78 (0.32)	10.9 (5.1)	10.1 (7.1)	0.47 (0.37)	0.19 (0.48*	0.01 (0.47)*

*p < 0.05

**Age and gender matched survival estimation based on the national Central Statistic Office’s database

***Respondents who expected to be alive at 70, 80 and 90 years age

### Current HRQOL and its relations with visual status

The average utility score by the EQ-5D was higher than with the TTO and the difference was 0.06 (*p* = 0.131). Happiness, as measured on a 0–10 VAS, was mean 6.3 (SD 2.2) (Table 2) The only statistically significant difference identified on all current HRQOL outcomes was for those who used home help in the last month.

**Fig. 1:**
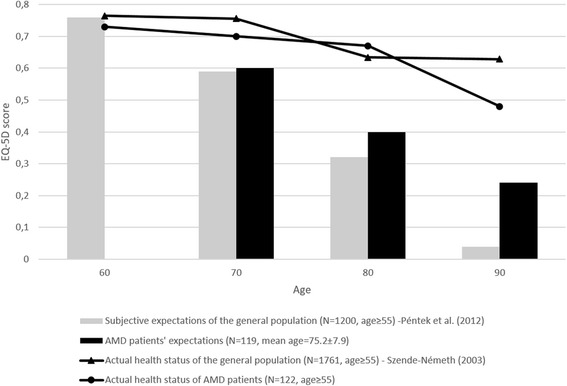
Comparison of subjective health-related quality of life (HRQOL) expectations in EQ-5D for older ages between AMD patients and the general population. General population and AMD patients between the age of 55–64, 65–74, 75–84 and 85–94 represent the age of 60, 70, 80 and 90 respectively. Expectations for age 60 of AMD patients are not depicted here since only three patients were < 60 years and answering expectation questions. Comparing future HRQOL expectations with actual health status of the age matched patients within the sample, actual EQ-5D scores exceeded the expectations (age of 60: 0.59 vs. 0.56, *P* > 0.05; age of 70: 0.73 vs. 0.38, *P* < 0.01). Sources: [13, 35]

The current EQ-5D score (mean 0.65, SD 0.34) was not statistically different compared to the age-matched general population’ values in Hungary (Fig. 1).

Correlations between EQ-5D score and visual acuity of left and right eyes were not significant, however, significant moderate correlation was found between visual acuity of the better eye and EQ-5D score (Table 3). The average EQ-5D score of patients with AREDS-4 in both eyes was lower than with AREDS-4 only in one eye (0.63 (SD 0.35) vs. 0.72 (SD 0.28)), the difference was not significant (*p* = 0.17).

**Table 3:** Correlations between subjective health expectations, happiness and continuous variables

Variables	EQ-5D utility (0–1)	TTO utility (0–1)	Happiness (0–10)	Expected remaining life years	Accuracy of life expectancy estimation	Expected EQ-5D scores for future ages		
						70y	80y	90y
Age		−0.032	−0.114	**−0.358**	**0.329**	**0.366**	**0.289**	0.052
ETDRS score in better eye	**0.241**	0.147	0.065	0.119	0.029	0.035	0.044	0.025
ETDRS score in worse eye	0.088	0.016	−0.022	0.078	−0.073	−0.013	−0.053	−0.157
VF-14 (0–100)	**0.373**	0.180	0.099	0.085	−0.048	0.191	0.151	0.168
TTO utility (0–1)	0.157	–	**0.286**	0.072	0.078	0.359	0.078	0.217
Current EQ-5D index score	–	0.157	0.146	0.112	0.098	**0.642**	**0.357**	**0.451**
Current EQ VAS (0–100)	**0.387**	0.144	**0.212**	0.182	0.162	**0.432**	**0.323**	**0.266**
Current general happiness VAS (0–10)	0.146	**0.286**	–	**0.298**	**0.221**	0.114	**0.267**	**0.532**

Bold: Correlation is significant at the *p* < 0.05 level

In contrast, direct utility scores measured by TTO did not correlate with visual acuity of left, right or the better eye (Table 3). However, significant differences were revealed between subgroups of AREDS-4 in both eyes and one eye (TTO: 0.68 vs. 0.84; *p* = 0.013). Patients who have already had antiVEGF therapy expected significantly better HRQOL for ages 80 and 90 compared to those who were only eligible but not yet experienced with this treatment. General happiness VAS did not correlate with visual acuity and did not differ between AREDS states either.

### Subjective life expectancy and its determinants

Subjective expectations regarding remaining life years were in line with patients’ gender- and age-matched statistical life expectancy (10.4 and 10.2, *p* = 0.980). Subgroup analysis by gender revealed that females underestimated their life-expectancy by 0.8 years, while males overestimated by 1.6 years (*p* = 0.051).

Age and self-reported general happiness showed significant moderate positive correlation, while EQ-5D score, EQ VAS, VF-14 and visual acuity showed no statistically significant correlation with subjective life-expectancy. Neither the number of expected remaining life years nor the accuracy of the life-expectancy estimation correlated with TTO results (Table 3).

Age was in moderate positive correlation with the accuracy of patients’ life expectancy estimation. Younger patients were more likely to underestimate whilst older ones to overestimate their life expectancy compared to the average statistical gender- and age-matched life expectancy (*R* = 0.329, *p* < 0.001). Also self-reported happiness was in positive association with the accuracy of patients’ life expectancy estimation, happier patients tend to overestimate their life expectancy (*R* = 0.221, *p* = 0.022).

### Subjective expectations for future HRQOL and its determinants

The majority of patients were older than 60 years, therefore HRQOL expectations related questions were relevant where patients were under age 70 (27%), 80 (54%) and 90 (57%). Patients expected a severe decrease of their HRQOL with age, from an average EQ-5D score of 0.60 at age 70 to 0.24 at age 90. Similar tendency was observed in a survey among the general population of age 55 and over, however, AMD patients seem to have somewhat more optimistic beliefs [13] (Fig. 1).

Among demographic variables, both age and gender were in significant association with future HRQOL expectations. Women expected for the future ages considerably lower HRQOL than men (Table 2). Age was in moderate positive correlations with future expectations, as older patients expected better HRQOL (Table 3). In other words, respondents with age closer to the questioned age expected better HRQOL.

Patients’ current HRQOL as measured by the EQ-5D and assessment about their happiness (on a 0–100 mm visual analogue scale) significantly correlated with future expectations. Higher score on patient reported happiness resulted in better self-estimated HRQOL for ages 80 and 90 years (Table 3). Results of linear regression analysis confirmed these findings indicating that age, current health status (EQ-5D and EQ VAS) and happiness VAS were significant determinants of future subjective health expectations (data will be sent on request).

## Discussion

This study provides data on subjective health expectations for the future in an AMD patient population who were eligible for or have experienced treatment with antiVEGF therapy. This is the first research in the literature to assess subjective health expectations of AMD patients and to explore the determinants. To study the relations between current visual status (AREDS, ETDRS), HRQOL and subjective expectations for future health, the VF-14 and two health state utility measures (the EQ-5D and the time trade-off) were applied and some further potentially influencing factors were also considered.

Our study showed that in this AMD population with high rate (76%) of biological drug treatment, patients’ EQ-5D scores were similar to those of age matched general population. This finding suggests that the generic health state measure EQ-5D is not able to capture the negative impact of AMD. The slight correlation between the EQ-5D score and visual acuity, and the statistically not significant difference of EQ-5D scores between AREDS states seem to strengthen this hypothesis. In contrast, the direct health status utility measurement, the TTO method, better distinguished between patients with different AREDS state (Tables 2 and 3). One possible reason of the different behaviour of the two health state utility measures and of their weak correlation is the difference in their anchor points, as EQ-5D was anchored to perfect health and TTO utility was anchored to perfect vision. Another disparity that might count is that the EQ-5D score presents societal values of health states whilst the TTO reflects patients’ evaluations. Our findings are in line with and confirm the conclusions of previous systematic reviews on utility measurement in wet AMD [19, 20]. Pearson et al. found that the TTO measurement is a better indicator of the impact of visual acuity on HRQOL than the EQ-5D. However, our study highlighted a new aspect, namely that both EQ-5D and TTO utilities were significantly lower in AMD patients who received help from others to perform everyday activities. Altogether 86% of patients using informal care had both eyes in AREDS-4, while 36% of patients having both eyes in AREDS-4 received informal care. EQ-5D utility difference was four times higher between informal care utiliser and non-utiliser subgroups than between AREDS subgroups (0.35 vs. 0.09). These data indicate that self-care ability was the determinant influential factor.

AMD patients expected a worse HRQOL for older ages than the general population in Hungary actually has in the respective ages. (Figure 1) Patients’ subjective expectations about their future HRQOL were associated with their current HRQOL as measured by the EQ-5D and also with their self-reported happiness. However, current health state as assessed by the TTO method did not correlate with subjective HRQOL expectations. Similarly to differences of current HRQOL between genders, men expected significantly better HRQOL for future ages than women. Likewise, informal care utilisation reflecting the dependence on others to perform everyday activities negatively influenced not only the current HRQOL but also future HRQOL expectations.

We compared AMD patients’ HRQOL expectations for the future to findings from the age-matched participants of a similar study among the Hungarian general population (Fig. 1) As future age increases the difference becomes greater, and for age 90 AMD patients expect by 0.2 better EQ-5D utility than the general population. However, in previously published studies patients with rheumatoid arthritis and psoriasis expected a sharp decrease of their HRQOL with age and their expectations were significantly more pessimistic than those of the general public [32, 34]. Higher age might be a possible explanation for AMD patients’ less pessimistic expectations, near future can be predicted easier. In our sample age was in a positive correlation with HRQOL expectations and mean age of AMD patients (75 years) was higher than either in rheumatoid arthritis, or in psoriasis samples or in the general population. Patients’ confidence in and experienced benefits with antiVEGF treatment might also have positive impact. Prospective studies are suggested to investigate further how patients’ experiences with antiVEGF treatment influence their subjective HRQOL expectations for future ages.

Besides current and expected future HRQOL, patients were asked about the age they expect to live. Subjective life-expectancy of AMD patients was consistent with the gender- and age-matched statistical life expectancy. This result suggests that antiVEGF treated AMD patients do not expect the disease having a negative impact on their length of life. Patients’ age and current happiness had significant influence on estimation accuracy. Older and happier patients expected more remaining life years than statistically predicted. The association between subjective life-expectancy and TTO was one of our points of interest, nevertheless we found no significant correlation between the two. We find important to note, however, that we used patients’ self-estimated longevity for the TTO. Given the similarities we found between subjective and statistical life-expectancies applying the latter in TTO studies seems to be a rational approach. Our results highlight that both current health state valuations and subjective health expectations are influenced various non-medical factors such as overall satisfaction with life (happiness) or dependence on others and are worthy to consider in future studies.

Comparing our results with other diseases, Rencz and colleagues observed similarly exact life expectancy estimation among psoriasis patients (*N* = 200, mean age = 50 ± 12) [32]. Péntek and colleagues found no significant difference either between the subjective life-expectancy of rheumatoid arthritis patients initiating biological treatment and actuarial life-expectancy (*N* = 92, mean age: 52 ± 12 years) [34]. However, an overestimation of subjective life-expectancy compared to statistical life-expectancy was observed in a large convenient sample of the general public in Hungary, and the difference was especially large among men [13]. It is hard to draw firm conclusions based on indirect comparisons. Nevertheless, these findings suggest that in chronic illnesses, including AMD, patients’ subjective expectations regarding remaining life years tend to lag behind compared to those of the general population.

Findings of this empirical investigation should be interpreted carefully given the limitations of the study. The study involved a convenience sample of AMD patients from two university based referral centres. The Hungarian statistical life expectancy data used for the comparisons were merely gender- and age-matched, although other socio-economic determinants, such as level of education, marital status and monthly income, might also have an impact on life expectancy. Due to the cross-sectional study design and heterogeneity of our sample in terms of biological treatment duration, we were not able to analyse the effects of antiVEGF therapy on subjective health expectations, a point that certainly deserves further investigation.

## Conclusions

Our findings suggest that AMD patients expect a significant deterioration of health with age and predict to have worse general health state in older ages than the general population actually has. Nevertheless, they expect to live as long as their age and gender matched counterparts in the general public. Worse subjective health expectations affect in particular the older patients in worse general health state. The most influential factor both on current HRQOL and future subjective health expectations was informal care. AMD patients requiring others’ help for self-care have worse HRQOL, expect shorter life and lower HRQOL for future ages. Patients’ happiness in general has deterministic role as well both in current health status utility evaluation and subjective health expectations. These two patient-related factors (informal care need and self-reported happiness) might be of relevance also in chronic diseases other than AMD and we encourage considering them in further health expectation studies. Results of our empirical investigation highlight the importance of including issues of long-term expectations in patient-clinician discussions in AMD care, to have a better knowledge and understanding of their perspective. Further longitudinal studies involving larger samples should bring more evidence on the link between subjective health expectations and actual health outcomes and the generalizability of the results.

## Additional file

Additional file 1: AMD questionnaire survey. The set of questions used in the survey to assess health-related quality of life, subjective health expectations and disease burden of patients with age-related macular degeneration treated with antiVEGF drugs. (DOC 188 kb)

## Abbreviations

AMD: Age-related macular degeneration
antiVGF: Anti-vascular endothelial growth factor
AREDS: Age-Related Eye Disease Study
EQ VAS: Health thermometer (visual analogue scale of the EQ-5D questionnaire)
EQ-5D: Health status questionnaire
ETDSR: Early Treatment Diabetic Retinopathy Study
HRQOL: Health-related quality of life
QALY: Quality-adjusted life year
TTO: Time trade-off
VAS: Visual analogue scale
VF-14: Visual Function Index-14

## References

[1] Boyers LN, Karimkhani C, Hilton J, Richheimer W, Dellavalle RP (2015). Global burden of eye and vision disease as reflected in the Cochrane database of systematic reviews. JAMA Ophthalmol.

[2] Cimarolli VR, Casten RJ, Rovner BW, Heyl V, Sorensen S, Horowitz A (2016). Anxiety and depression in patients with advanced macular degeneration: current perspectives. Clin Ophthalmol.

[3] Dawson SR, Mallen CD, Gouldstone MB, Yarham R, Mansell G (2014). The prevalence of anxiety and depression in people with age-related macular degeneration: a systematic review of observational study data. BMC Ophthalmol.

[4] Freund KB, Korobelnik JF, Devenyi R, Framme C, Galic J, Herbert E, Hoerauf H, Lanzetta P, Michels S, Mitchell P (2015). Treat-and-extend regimens with anti-VEGF agents in retinal diseases: a literature review and consensus recommendations. Retina.

[5] Schmidt-Erfurth U, Chong V, Loewenstein A, Larsen M, Souied E, Schlingemann R, Eldem B, Mones J, Richard G, Bandello F (2014). Guidelines for the management of neovascular age-related macular degeneration by the European Society of Retina Specialists (EURETINA). Br J Ophthalmol.

[6] Wong TY, Chakravarthy U, Klein R, Mitchell P, Zlateva G, Buggage R, Fahrbach K, Probst C, Sledge I (2008). The natural history and prognosis of neovascular age-related macular degeneration: a systematic review of the literature and meta-analysis. Ophthalmol.

[7] Veenhoven R (1991). Is happiness relative?. Soc Indic Res.

[8] Yew-Kwang NG (1996). Happiness surveys: some comparability issues and an exploratory survey based on just perceivable increments. Soc Indic Res.

[9] van de Wetering E, van Exel N, Brouwer W (2010). Piecing the jigsaw puzzle of adolescent happiness. J Econ Psychol.

[10] Senra H, Ali Z, Balaskas K, Aslam T (2016). Psychological impact of anti-VEGF treatments for wet macular degeneration-a review. Graefes Arch Clin Exp Ophthalmol.

[11] Boyle J, Vukicevic M, Koklanis K, Itsiopoulos C (2015). Experiences of patients undergoing anti-VEGF treatment for neovascular age-related macular degeneration: a systematic review. Psychol Health Med.

[12] Brouwer WB, van Exel NJ (2005). Expectations regarding length and health related quality of life: some empirical findings. Soc Sci Med.

[13] Pentek M, Brodszky V, Gulacsi AL, Hajdu O, van Exel J, Brouwer W, Gulacsi L (2014). Subjective expectations regarding length and health-related quality of life in Hungary: results from an empirical investigation. Health Expect.

[14] Kymes Steven M (2009). The cost-effectiveness of treatment of age-related macular degeneration: a review. Minerva Med.

[15] Boncz I, Sebestyen A (2006). Financial deficits in the health services of the UK and Hungary. Lancet.

[16] Brown MM, Brown GC, Lieske HB, Lieske PA (2014). Financial return-on-investment of ophthalmic interventions: a new paradigm. Curr Opin Ophthalmol.

[17] Shah AR, Williams GA (2016). Regulatory and economic considerations of retinal drugs. Dev Ophthalmol.

[18] Hodgson N, Wu F, Zhu J, Wang W, Ferreyra H, Zhang K, Wang J. Economic and quality of life benefits of anti-VEGF therapy. Mol Pharm. 2016. 10.1021/acs.molpharmaceut.5b00775 26836112

[19] Pearson I, Rycroft C, Irving A, Ainsworth C, Wittrup-Jensen K (2013). A systematic literature review of utility weights in wet age-related macular degeneration. J Med Econ.

[20] Poku E, Brazier J, Carlton J, Ferreira A (2013). Health state utilities in patients with diabetic retinopathy, diabetic macular oedema and age-related macular degeneration: a systematic review. BMC Ophthalmol.

[21] Arnesen T, Trommald M (2005). Are QALYs based on time trade-off comparable?--a systematic review of TTO methodologies. Health Econ.

[22] Attema AE, Edelaar-Peeters Y, Versteegh MM, Stolk EA (2013). Time trade-off: one methodology, different methods. Eur J Health Econ.

[23] Rappange DR, Brouwer WB, van Exel J (2016). Rational expectations? An explorative study of subjective survival probabilities and lifestyle across Europe. Health Expect.

[24] van Nooten FE, van Exel NJ, Eriksson D, Brouwer WB (2016). "back to the future": influence of beliefs regarding the future on TTO answers. Health Qual Life Outcomes.

[25] EuroQol G (1990). EuroQol--a new facility for the measurement of health-related quality of life. Health Policy.

[26] Cantrill HL (1984). The diabetic retinopathy study and the early treatment diabetic retinopathy study. Int Ophthalmol Clin.

[27] Clemons TE, Chew EY, Bressler SB, McBee W (2003). age-related eye disease study research G: National eye Institute visual function questionnaire in the age-related eye disease study (AREDS): AREDS report no. 10. Arch Ophthalmol.

[28] Mackenzie PJ, Chang TS, Scott IU, Linder M, Hay D, Feuer WJ, Chambers K (2002). Assessment of vision-related function in patients with age-related macular degeneration. Ophthalmol.

[29] Stalmeier PF, Goldstein MK, Holmes AM, Lenert L, Miyamoto J, Stiggelbout AM, Torrance GW, Tsevat J (2001). What should be reported in a methods section on utility assessment?. Med Decis Mak.

[30] Torrance GW (1987). Utility approach to measuring health-related quality of life. J Chronic Dis.

[31] Torrance GW (1986). Measurement of health state utilities for economic appraisal. J Health Econ.

[32] Rencz F, Hollo P, Karpati S, Pentek M, Remenyik E, Szegedi A, Balogh O, Heredi E, Herszenyi K, Jokai H (2015). Moderate to severe psoriasis patients' subjective future expectations regarding health-related quality of life and longevity. J Eur Acad Dermatol Venereol.

[33] Hungarian Central Statistical Office, Statistics Database. Available at: http://www.ksh.hu/. Accessed 11 Jan 2015.

[34] Pentek M, Gulacsi L, Rojkovich B, Brodszky V, van Exel J, Brouwer WB (2014). Subjective health expectations at biological therapy initiation: a survey of rheumatoid arthritis patients and rheumatologists. Eur J Health Econ.

[35] Szende A, Nemeth R (2003). Health-related quality of life of the Hungarian population. Orv Hetil.

