# Supplementary material for: Subjective health expectations of patients with age-related macular degeneration treated with antiVEGF drugs
Source: BMC Geriatr. 2017 Oct 10;17:233. doi: 10.1186/s12877-017-0619-9 (PMC5635533; doi:10.1186/s12877-017-0619-9)
Supplement: Additional file 1: — AMD questionnaire survey. The set of questions used in the survey to assess health-related quality of life, subjective health expectations and disease burden of patients with age-related macular degeneration treated with antiVEGF drugs. (DOC 188 kb) [file 12877_2017_619_MOESM1_ESM.doc]

**Health-related quality of life and disease burden of patients with age-related macular degeneration in Hungary**

**Questionnaire survey**

**2012.**

**I. Demography**

**Date:** year  month  day

1. **Gender (mark with X)**

Male

Female

1. **Date of birth** (year)
2. **Starting date of vision impairment** year  month
3. **Houshold situation**

Lives alone

Lives with others

1. **Highest educational level (mark with X)**

Primary school

Secondary school

College

University

1. **Place of living**

Budapest

County capital

Other city

Village, other

1. **Net monthly income? (mark with X)**

0 – 75 000 HUF / month

75 500 – 150 000 HUF / month

150 500 – 250 000 HUF / month

250 500 – 350 000 HUF / month

350 500 – 450 000 HUF / month

550 000 or more HUF / month

**II. Employment status**

1. **Are you employed (doing a payed job)?**

Yes

No

**If no, please go to question Nr. 10.**

**8. a.) If you work:**

Do you work in a full-time job? Yes  No

Do you work in a part-time job? Yes  No

**8. b.) If you work in a part-time job:**

Do you work in part-time due to your macular disease? Yes  No

How many hours do you work per week? ______ hours/week.

**9. How many times your macular disease affected your payed work in the past 12 months?**

You were on sick-leave due to macular disease  days

Your were on payed holiday due to your macular disease  days

Your were on unpayed holiday due to your macular disease  days

None of the above list happened

1. **If you do not work in a payed job:**

on disability pension

retired

unemployed

did not work before or left job due to pther reasons

1. **If you are on disability pension:**

Are you on disability pension due to your macular disease? Yes  No

Since when?  (year)

Current level of disability:  %

**III. Heathcare resource utilisations**

1. **Have you visited your GP due to your macular disease in the past 12 months?**

Yes  No

If yes, how many times?

1. **Have you visited an ophthalmologist due to your macular disease in the past 12 months?**

Yes  No

If yes, how many times?

1. **Have you been admitted to hospital due to your macular disease in the past 12 months?**

Yes  No

If yes, how many times?

# How many days did you spent in the hospital due to macular disease (past 12 months)?:

1. **Please indicate which therapies you had in the past 12 months!**

Photocoagulation  How many times?

Photodynamic therapy (PDT)  How many times?

Injection  How many times?

Did not have treatment

1. **Did you visit a specialist in the private sector (not reimbursed) due to your macular disease in the past 12 months?**

Yes  No

**If yes, how many times?**

Private care visits ⬜ ⬜ times in the past 12 months.

How much did you pay for these visits in the past 12 months?

………………….……. HUF.

1. Diy you fall in the past 12 months?

Yes  No

1. If yes, did you have a bone fracture?

Yes  No

18.a) Type of the fracture? Wrist  Hip  Other:..………………….

18.b) Did you have medical treatment due to the fracture? Yes  No

18.c) Were you admitted to hospital due to the fracture? Yes  No

1. **What kind of aids and devices did you use** **due to your macular disease in the past 12 months?**

**(You can indicate more than one, mark with X)**

R = reimbursed aids and devices

P = out-of-pocket payment for the aids and devices

R P Quantity (pieces)

Glasses

White cane

Binocular glasses

Magnifying glasses

Other: …………………………………………    …………….…………………………..

…………….…………………………..

…………….…………………………..

**How much did you spent in the past 12 months on devices that are not reuimbursed?** ………………….………………...HUF

1. **Do you use eye drops?**

Yes  No

If yes, are you able to apply it alone or you need help for that?

Alone  With help from others

1. **How do you travel to the ophthalmology centre? (mark with X)**

With whom you travel? With what you travel?

Alone  Public transportation

Accompanied by someone  Bus

Train

Car

Ambulance

Walk

1. **How many times did you use the following services due to your macular disease care (treatments, check-ups) in the past 12 months:**

Ambulance

Travel voucher

Never

1. How far is the ophthalmology centre (where you are regularly get care) from your home?

0 – 25 km

25 – 50 km

50 – 100 km

100 – 200 km

more than 200 km

**IV. Help from others for everyday activities**

1. **Did you receive help from others due to your macular disease in the past 1 month? (eg. for houskeeping, shopping, self-care)**

Yes No

**24.a) If yes, how much time per week from family members or other non-professionals?**

times per week  hours per day.

**24.b) If yes, how much time per week from payed professionals?**

times per week  hours per day.

**24.c) Who helped you and in which activities (mark with X)? Please indicate the number of hours**

Friend or family Payed member professional

Housekeeping …….hours/week …….. hours/week

Other home works (pl. shopping) …….hours/week …….. hours/week

Self-care (eg. dressing, washing) …….hours/week …….. hours/week

Total …….hours/week …….. hours/week

If you had payed help, how much did you pay for that in the past 1 month? …………………….HUF

**24.d) Did you have help from social care services?**

Yes  No

If yes, how many days per months in average?  days/month

1. **Do you have special licence for parking?**

Yes  No

**V. EQ-5D-3L questionnaire**

EuroQol G. EuroQol--a new facility for the measurement of health-related quality of life. Health Policy 1990;16:199-208.

**VI. VF-14-QOL questionnaire**

Mackenzie PJ, Chang TS, Scott IU, Linder M, Hay D, Feuer WJ, et al. Assessment of vision-related function in patients with age-related macular degeneration. Ophthalmology 2002;109:720-729.

**VII. Subjective health expectations**

**In the next questions we will ask you about your subjective expctations regarding your future health.**

People generally have some expectations about their future health.

In the next questions we ask you to indicate the health status you expect for yourself at ages **60, 70, 80 and 90** years.

**If you are older, than please please skip to the next question.**

**Mark your response with X**

**I think at the age of 60 I will have… (Please mark your response)**:

| No |  | Some |  | Major |  |
| --- | --- | --- | --- | --- | --- |
|  |  |  |  |  | problems with walking about. |
| No |  | Some |  | Major |  |
|  |  |  |  |  | problems with washing and dressing. |
| No |  | Some |  | Major |  |
|  |  |  |  |  | problems performing usual activities. |
| No |  | Some |  | Severe |  |
|  |  |  |  |  | pain or discomfort. |
| No |  | Some |  | Severe |  |
|  |  |  |  |  | anxiety or depression. |

**I think at the age of 70 I will have… (Please mark your response):**

| No |  | Some |  | Major |  |
| --- | --- | --- | --- | --- | --- |
|  |  |  |  |  | problems with walking about. |
| No |  | Some |  | Major |  |
|  |  |  |  |  | problems with washing and dressing. |
| No |  | Some |  | Major |  |
|  |  |  |  |  | problems performing usual activities. |
| No |  | Some |  | Severe |  |
|  |  |  |  |  | pain or discomfort. |
| No |  | Some |  | Severe |  |
|  |  |  |  |  | anxiety or depression. |

**I think at the age of 80 I will have… (Please mark your response):**

| No |  | Some |  | Major |  |
| --- | --- | --- | --- | --- | --- |
|  |  |  |  |  | problems with walking about. |
| No |  | Some |  | Major |  |
|  |  |  |  |  | problems with washing and dressing. |
| No |  | Some |  | Major |  |
|  |  |  |  |  | problems performing usual activities. |
| No |  | Some |  | Severe |  |
|  |  |  |  |  | pain or discomfort. |
| No |  | Some |  | Severe |  |
|  |  |  |  |  | anxiety or depression. |

**I think at the age of 90 I will have… (Please mark your response):**

| No |  | Some |  | Major |  |
| --- | --- | --- | --- | --- | --- |
|  |  |  |  |  | problems with walking about. |
| No |  | Some |  | Major |  |
|  |  |  |  |  | problems with washing and dressing. |
| No |  | Some |  | Major |  |
|  |  |  |  |  | problems performing usual activities. |
| No |  | Some |  | Severe |  |
|  |  |  |  |  | pain or discomfort. |
| No |  | Some |  | Severe |  |
|  |  |  |  |  | anxiety or depression. |

**How long do you expect to live?**

| I expect to live until the age of ….., in other words I expect to live further …. life years. |
| --- |

**How many years would you sacrify of your remaining life in return of perfect vision?**

|  | years. |
| --- | --- |

**Please evaluate your happiness!** Please indicate on the scale below how much happy you feel yourself in general. The ’0’ means very unhappy and ’10’ means very happy.

0 1 2 3 4 5 6 7 8 9 10

very unhappy very happy

**PLease check whether you answered all the questions**

**Thank you for helping our work by completing this survey.**

**VII. Questions for the ophthalmologist**

1. **Type of care at the time of the survey (Mark with X)**

Outpatient care  Inpatient care  One-day care

1. **When was the patient diagnosed with AMD?**

rigth eye  year  month

left eye  year  month

1. **Was the visual acuity impairment of the currently treated eye diagnosed in that date?**

Yes  No

If not, then when was the currently treated eye diagnosed?

year  month

1. **Visual acuity of the eyes**

Right eye, visual acuity:………………….. Is this eye currently treated? Yes No

Left eye, visual acuity:…………………… Is this eye currently treated? Yes No

1. **Since when is the patient treated (cared) in this centre?**

year  month

1. **Please indicate the AREDS state of the patient for each eye (AREDS 1-2-3-4)**

Right eye:………………….. Left eye:…………………..

1. **Have the currently treated eye worsen since the last visit?**

Yes  No

If yes, how much?  letters deterioration

Was the worsening of 15 ETDRS? Yes  No

1. **Please indicate the therapies the patient got in the past 12 months**

**Frequency Quantity**

Drugs: …………………… ……/…… ……….. mg/ml/piece/inj

…………………… ……/…… ……….. mg/ml/piece/inj

…………………… ……/…… ……….. mg/ml/piece/inj

Photocoagulation ……/…… ……….. mg/ml/piece/inj

Photodynamic therapy (PDT) ……/…… ……….. mg/ml/piece/inj

Pegaptanib ……/…… ……….. mg/ml/piece/inj

Ranibizumab ……/…… ……….. mg/ml/piece/inj

Bevacizumab ……/…… ……….. mg/ml/piece/inj

……………………. ……/…… ……….. mg/ml/piece/inj

……………………. ……/…… ……….. mg/ml/piece/inj

1. **Therapies the patient currently gets**

Since when? Quantity

Drugs: …………………..… …………………. ……………………

…………………… …………………. ……………………

…………………… …………………. ……………………

Photocoagulation …………………. ……………………

Photodynamic therapy (PDT) …………………. ……………………

Pegaptanib …………………. ……………………

Ranibizumab …………………. ……………………

Bevacizumab …………………. ……………………

……………………. …………………. ……………………

……………………. …………………. ……………………

1. **How many times did the patient have the following diagnostics in the past 12 months due to his/her AMD?**

How many times?

Fluorescein angiography (FLAG)

Optical Coherence Tomography (OCT)

Visual acuity

Dilated eye exam

Fundoscopy

Refraction

Tonometry

1. **Why was the therapy stopped?**

Allergic reactions

Intolerance

Other: ………………………

1. **Comorbidities**

Parkinson’s disease

Diabetes

Depression

Hypertension

Other:…………………………….

…………………………….

1. **Name, date, signature**

………………………………………………………………………………………

**Thank you!**
